# Adverse effects of routine bovine health treatments containing triclabendazole and synthetic pyrethroids on the abundance of dipteran larvae in bovine faeces

**DOI:** 10.1038/s41598-019-40800-6

**Published:** 2019-03-13

**Authors:** Gillian Gilbert, Fiona S. MacGillivray, Helen L. Robertson, Nicholas N. Jonsson

**Affiliations:** 1 Royal Society for the Protection of Birds, Centre for Conservation Science, 10 Park Quadrant, Glasgow, G36BS Scotland UK; 2 Royal Society for the Protection of Birds, Bushmill Cottage, Gruinart, Bridgend Isle of Islay, Argyll, PA44 7PR Scotland UK; 3 University of Glasgow College of Medical, Veterinary and Life Sciences, 464 Bearsden Rd Glasgow, G61 1QH Scotland, UK

## Abstract

Macrocyclic lactone treatments for livestock can have detrimental effects on the arthropod populations in livestock faeces. For the last twenty years, avoidance of these products has been a standard recommendation on livestock farms that are managed for wildlife by the Royal Society for Protection of Birds (RSPB). However, the continued decline in the populations of birds (in particular the red-billed chough *Pyrrhocorax pyrrhocorax*) that are dependent on dung invertebrates on islands in the Inner Hebrides of Scotland prompted us to investigate the effects of livestock treatments that are commonly used on these islands. We conducted a replicated field plot study over two years to quantify the effects of livestock treatments containing copper, deltamethrin and triclabendazole on invertebrate density in pooled, artificial faecal pats on the island of Islay. We found that the density of arthropod larvae was significantly reduced by the triclabendazole and deltamethrin treatments in both years and by as much as 86% when the treatments were combined. Copper-containing boluses did not consistently affect abundance of arthropod larvae. These results suggest that veterinary treatment of livestock might contribute to a reduction in the food supply of chough.

## Introduction

Islay, Oronsay and Colonsay are the southernmost islands of the archipelago known as the Inner Hebrides of Scotland. They provide habitat for many permanent and migratory species of birds and are the sites of the only remaining breeding colonies of red-billed chough (chough, *Pyrrhocorax pyrrhocorax*) in Scotland. The islands are characterised by very heterogeneous geology, soil, habitat and land-use patterns. Much of the land is grazed by sheep and cattle in low-intensity, ‘high nature value’ farming systems^1^. This system of grazing, which fosters a combination of short grassland vegetation and a rich soil, dung and epigeic invertebrate fauna, is ideal for chough. However, in recent years the population of chough on Islay, Oronsay and Colonsay has been in severe decline, considered likely to be due to declining feed resources^2,3^.

More than twenty years ago, the macrocyclic lactone (ML) cattle treatments, avermectins were found to have lethal and sub-lethal effects on arthropods^4–6^, which can persist^7^ for up to a month after administration as injectable or topical formulations^8^. Ivermectin in faeces was considered to be likely to reduce the availability of food for chough^9–11^. Although farm and landscape scale studies conducted by the RSPB over 2 years found inconsistent evidence of reductions in dung insect populations that could be associated with routine use of MLs^12,13^, these products have since the late 1990s been recommended not to be used on farms where chough feed^14^. The recommendation became policy on farms owned and managed by RSPB on Islay, Oronsay and Colonsay, and most private livestock farmers on the islands are aware of and tend to comply with the recommendation (Gilbert, Jonsson, McGillivray – unpublished data). In the last 20 years the livestock treatments used commonly in the UK (and elsewhere) and their frequency of application have changed^15^. Although there has been a general reduction in the burden of livestock disease as a result of more effective drugs and vaccines and improvements in diagnosis^15^, there is evidence that climate change, especially elevated temperature, has significantly increased the level of challenge by helminth parasites in the UK, particularly in south-western Scotland^16,17^. There is clear evidence of increased frequency of resistance to anthelmintic treatments in parasites of cattle^18^, and emerging resistance to parasiticides often leads to increased frequency of application with the same products^19^. The important parasitic problems of sheep and cattle on Islay are broadly consistent with the rest of the western United Kingdom and include liver fluke (a trematode - *Fasciola hepatica*) and the tick *Ixodes ricinus*, both of which are shared by sheep and cattle. Liver fluke infection directly causes deaths in sheep and cattle, and the pathogens that *Ixodes ricinus* ticks transmits include louping ill virus, *Anaplasma phagocytophilum* and *Staphylococcus spp*., all of which can be fatal. Commercially available treatments for liver fluke include albendazole, closantel, and nitroxynil, which are effective against adult fluke, and triclabendazole, which is effective against all stages of the fluke. For the control of ticks, the most commonly used treatments are synthetic pyrethroids, mostly cypermethrin, alpha-cypermethrin, deltamethrin and permethrin.

Whereas MLs have been the focus of most scientific attention in relation to faecal invertebrates, there is growing evidence that a range of other animal treatments can have deleterious effects^11,20–24^. Triclabendazole (TCBZ) is a member of the benzimidazole family of anthelmintics, which is rapidly removed from the blood stream, metabolised in the liver and excreted in the bile as the active compound triclabendazole sulfoxide (TCBZ-SO) and the inactive metabolite triclabendazole sulfone (TCBZ-SO_2_), with subsequent excretion in the faeces^25^. A significant adverse effect *in vitro* of low concentrations of TCBZ (0.001 to 0.1%) on the survival and development of the greater wax moth *Galleria mellonella* has recently been demonstrated^26^. However, it is not clear how these concentrations relate to those that are present in the faeces of treated animals and whether any detectable effect should be expected after routine treatment of livestock. Deltamethrin is a synthetic pyrethroid (SP) product, which is widely applied on cattle, sheep and lambs to control arthropod ectoparasites. On Islay, Oronsay and Colonsay, the product is commonly used for the control of *Ixodes ricinus* ticks. Ninety-five percent of any dose of deltamethrin applied by pour-on is excreted in the faeces, mostly in metabolised but active form^27^. Wardhaugh *et al*. conducted bioassays in faeces^28^, the results of which led them to suggest that under Australian conditions, one treatment in a year with a deltamethrin based insecticide could cause up to 75% reduction in dung beetle activity by the end of a season and multiple treatments might drive local populations towards extinction. Similar studies in Europe^20,29^ and Africa^30^ report variation in the negative effects of deltamethrin depending on the sensitivities of the arthropod species in question. These previously published studies on deltamethrin and triclabendazole were bioassays, conducted in laboratories and did not examine all the species that are known to be important in the UK. It is not known if the results can be extrapolated to the field conditions that are important to a feeding chough, which are adapted to breaking apart older more friable dung containing developing larvae. However, they strongly suggest that the use of these products on livestock might be important factors in the ecology of invertebrates that provide a feed source for chough.

The aim of this study was to experimentally quantify the effects of commonly used veterinary parasiticide treatments on invertebrate density in grasslands on which chough depend as a foraging habitat. The study was designed to provide the rigour of a controlled and replicated design but under conditions that matched the natural environment for the invertebrates and using the same, commercially available products exactly as they are used by farmers in that environment.

## Materials and Methods

All control and treatment faeces was collected in 2014 and 2015 from the same small herd of between 10 and 12 highland cattle from the Oa nature reserve on Islay, Scotland (55.59825N, 6.31637W), managed by the Royal Society for the Protection of Birds (RSPB). The cattle were in the same fields during the period when dung was collected in both years, and herd management was the same in both years. The cattle were kept outside during the winter on a hillside of rough grazing, during which time no veterinary treatments were administered. In April of both years, the cattle were moved into improved pasture fields that were known to be favoured by foraging chough, adjacent to the rough grazing area.

### Faecal collection

**Figure 1:**
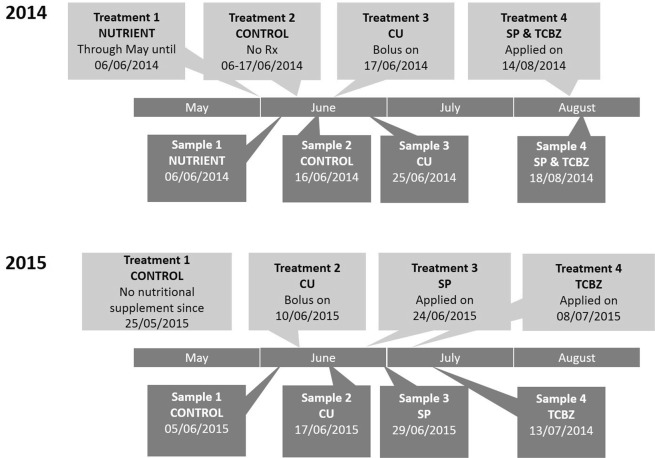
Timeline of the application of treatments and collection of samples in 2014 and 2015. NUTRIENT is a cereal-based supplementary feed source available to the cattle; CU is a slow release (6 month) copper and iodine bolus; SP is a synthetic pyrethroid spot-on treatment containing deltamethrin; TCBZ is a drench treatment containing triclabendazole.

The dates of administration of treatments and collection of faeces are shown in Fig. 1. Samples were collected 7 days after administration of the Cu-containing bolus; five days after administration of SP and TCBZ together, and 4 days after SP or TCBZ separately. Cattle were observed and samples of faeces were collected within minutes after defecation. At least 30 litres of faeces from each treatment was collected, (except for samples collected in 2014 after the NUTRIENT treatment, of which only 22 litres was collected, enough to form 22 experimental pats rather than the required 27). Faeces was combined in large 30 litre covered plastic buckets, coded, frozen and kept under constant −20 °C, within six hours after collection. This process is known to kill invertebrates in the dung, but subsequent colonisation and reproduction by dung invertebrates when the dung is redeployed in the field is unaffected^23,31^.

No ethical approval was necessary for any administration of treatments to cattle used in this study. All treatments relevant to this study would have happened anyway, these were routine treatments given at routine times. It was necessary for the purposes of our study, that the results reflect what happens in a normal or completely routine farming operation.

### Cattle treatments

#### *Control* (CONTROL)

In 2014 and 2015 control dung was taken after a period of at least 6 months since administration of any medical treatments and 10 days after withdrawal of a nutritional supplement. The herd had access to a nutritional supplement (detailed below and included as a treatment) up to 10 days before control dung was taken. In accordance with their normal routine, apart from the nutritional supplement, the herd had no medical treatments for at least 6 months before control dung was taken.

#### *Nutrient supplement* (NUTRIENT, 2014 only)

Suckler cow rolls. These were a commonly used, commercially available cereal-based energy and protein supplement fed as a complement to pasture. Their composition is: Oil 4%, Fibre 12.5%, Moisture 14%, Selenium 0.5 mg/kg, Magnesium 2.25%, Protein 16%, Ash 16%, Sodium 0.8%, Copper 41 mg/kg, with vitamin and trace element additives.

#### *Copper bolus* (CU)

Tracesure Cu/I (Animax, UK), a slow release reticuloruminal bolus for cattle to supplement key trace elements over 6 months. Each bolus supplies 19 mg of Iodine, 3 mg of cobalt, 3 mg of selenium and 13 mg absorbable copper per day by slow release from the reticulorumen for 5–6 months.

#### *Triclabendazole* (TCBZ)

Fasinex 240 (Elanco), an anthelmintic treatment for *Fasciola hepatica* (liver fluke) infections in cattle. It contains 24% w/v triclabendazole as the active ingredient, and is administered orally as a drench.

#### *Synthetic pyrethroid spot-on* (SP)

Spot On (Coopers), a ready-to-use topical ectoparasiticide containing deltamethrin 1% w/v as the active ingredient, applied to the skin of the animal, on the mid-line of the back at the wither.

### Dung colonisation study sites

Three experimental grids were laid out at each of three sites, all of which were known to attract foraging chough. Each grid was located to represent the topographical diversity of the site in areas where chough were likely to feed, and where cattle and sheep were present, together with dung of different ages in close proximity, suggesting that colonisation by dung invertebrates would be likely. The three sites were as follows: 1. Ardnave (55.886535N, 6.333182W): a short-grazed sand–dune pasture with a constant presence of cattle. The grids were placed on the eastern and western sides of the peninsula. 2. Smaull Farm (55.825836N, 6.449569W) a pasture-dominated farm on the western coast of Islay. 3. The Oa: already described above, as the site of treatment of animals and collection of faeces.

Samples of dung from each cattle treatment (see Fig. 1 for timeline) were formed into 1-litre volume experimental ‘faecal pats’. Each of the grids, of which there were a total of nine in each year, contained 12 experimental faecal pats. Each grid had three rows of four experimental faecal pats, rows being 2 m apart and each row contained four faecal pats at 2 m spacings, each comprising one of the four treatments, placed in random sequence within the row. Sequence of position within the row was determined using a random number generator. Each experimental faecal pat was individually protected from birds by a wire cage secured into the ground. For the missing NUTRIENT pats in 2014, empty cages were set up in the locations where the NUTRIENT dung should have been. In 2014 there was a total of 103 experimental faecal pats, in 2015 there were 108 experimental faecal pats. In both years, the array of experimental faecal pats described above was established on 3 September. In both years, we avoided setting out and sampling the grids in wet or windy conditions, post hoc weather data^32^ for that day and for the whole sampling period are presented.

### Dung colonisation data collection

Immediately after each grid was established, the experimental faecal pats were observed for 15 minutes and all arthropods that were seen to be attracted to the surface of the faeces were recorded. At one, two and four week intervals thereafter, two standardised 6.5 cm (diameter) cores were sampled from all the experimental faecal pats, drilling through both the faeces and the upper 5 cm of soil. In weeks one, two and four, all samples were processed in the field; invertebrates in the dung and soil were separated and identified to the level of family and the cores were replaced in the experimental faecal pat. In week four, in addition to the standard core samples, the rest of the experimental faecal pat was also sampled. These sampling methods were designed to quantify the invertebrate species that would be likely to be preyed on by chough. Chough will pick through faecal pats that are at any stage of decay, but most profitable faecal pats are ‘medium to old’^33^. Our main sampling at 4 weeks was intended to count maturing *Aphodius* larvae. The sampling at one and two weeks was intended to count the faster maturing dipteran larvae.

### Comparison of experimental faecal pats with naturally occurring faecal pats

Twenty-seven naturally occurring faecal pats, which were estimated to be between one and four weeks old, were located within 50 m of the experimental grids at each site. These naturally occurring faecal pats were examined using the same method as for the experimental faecal pats, and the invertebrate densities of these cores were compared with those of the two-week-old cores from the control experimental faecal pats. Except at the Oa, the cattle that had produced the naturally occurring faecal pats were not part of the experiment and would have been subject to their normal veterinary treatment regime, details of which were not available to us.

### Data analysis

We tested for differences in abundance of invertebrates found in experimental faecal pats that were obtained from cattle that were untreated or treated with livestock health products. In 2014 treatments were (i) CONTROL, (ii) NUTRIENT, (iii) COPPER, and (iv) combined TCBZ and SP parasiticide treatments, and in 2015 were (i) CONTROL, (ii) COPPER (iii) TCBZ, and (iv) SP. The abundance data were analysed using generalised mixed models assuming a Poisson distribution and a log-link function. Degrees of freedom were corrected by the Kenward-Roger method, and overdispersion was corrected for using Pearson chi-square divided by degrees of freedom. Response variables for the abundance models were: (i) number of arthropod larvae, (ii) number of arthropod adults, and (iii) number of earthworms. Fixed effects were treatment, and for the cores sampled in weeks 1, 2 and 4, week was included as a fixed effect, together with the interaction between treatment and week. In all analyses, the two core samples taken from each faecal pat on each occasion were combined and treated as a single sample. Random effects included site, grid, column-in-grid, row-in-grid and individual experimental faecal pat. Fixed effect significance was examined by type III sums of squares and least means squares comparisons between treatments. Where degrees of freedom refer to factor levels this is stated as DF_level_, otherwise degrees of freedom are reported with numerator, denominator (F tests) or denominator (least squares means differences as t tests), with fractional denominator estimates from the Kenward Rogers method. Random effects were examined by the Wald test on their parameter estimate outcomes. If the random effect variable exhibited no variation, or was not significant in the model, it was removed from the model and the analysis. Modelling was carried out using SAS PROC GLIMMIX^34^.

## Results

### Initial attractiveness of the dung

429 and 948 adult arthropods were attracted to the surfaces of all the experimental faecal pats in the first 15 minutes of observation in 2014 and 2015 respectively. Most of these adults were yellow dung flies *Scathophaga stercoraria* (357 in 2014 and 921 in 2015). There was a greater variety of other invertebrates present in 2014 than in 2015: 28 beetles (*Coleoptera*), of *Aphodius*, *Hydrophillidae* and *Staphylinidae* and 45 other flies (*Diptera*) of *Muscidae*, *Stratiomyidae*, *Bibionidae*, (*Sepsidae* and *Lauxaniidae*), and *Anisopodidae*; whereas in 2015, apart from yellow dung flies, there were 27 other flies of *Muscidae*, *Sepsidae* and *Lauxaniidae*. The initial attractiveness of the faeces, as indicated by the number of arthropods counted on its surface within 15 minutes after presentation did not differ according to the treatments given to cattle within either year (Table 1, in which the relevant results are for the whole surface sample at week 0). The weather on the 3^rd^ of September in both years was recorded in the field as “dry, mild and calm”. Post hoc weather data^32^ for Islay on that day was as follows, mean temperature °C: 2014 (14.1, 13–18), 2015 (11.7, 10–15); relative (%) humidity 2014 (81, 56 minimum), 2015 (82, 72 minimum); mean wind speed m/s 2014 (3.6), 2015 (7.8), with no rainfall during the setting out period.

**Table 1:** Generalised linear mixed model results of the effect of cattle treatments on the abundance of invertebrates in experimental faecal pats in random block grids, with four treatments each year, including a control.

Year	Sample	Week	Larvae or Adult	F	P	DF
2014	whole surface	0	Adults	2.54	0.0616	3, 84.8
2015	whole surface	0	Adults	1.17	0.1307	3,56.7
2014	Cores	1,2,4	Larvae	6.85	0.0003	3, 101.5
2015	Cores	1,2,4	Larvae	5.65	0.0013	3,91.7
2014	Cores	1,2,4	Adults	1.25	0.291	3,312
2014	whole experimental pat	4	Larvae	20.06	<0.0001	3, 65.8
2015	whole experimental pat	4	Larvae	14.99	<0.0001	3,88.2
2014	whole experimental pat	4	Earthworms	5.26	0.003	3,52.2
2015	whole experimental pat	4	Earthworms	20.09	<0.0001	3,64.6
**Anova results comparing arthropod abundance between experimental control pats and ‘natural’ pats, where the ‘−’ signifies lower abundance in ‘natural’ pats**.						
2014	Cores	2	Adults	−5.8	0.019	1,26
2014	Cores	2	Larvae	−10.38	0.0022	1,26
2015	Cores	2	Adults	−0.23	0.666	1,26
2015	Cores	2	Larvae	−3.2	0.079	1,26

### Arthropod larvae

**Figure 2:**
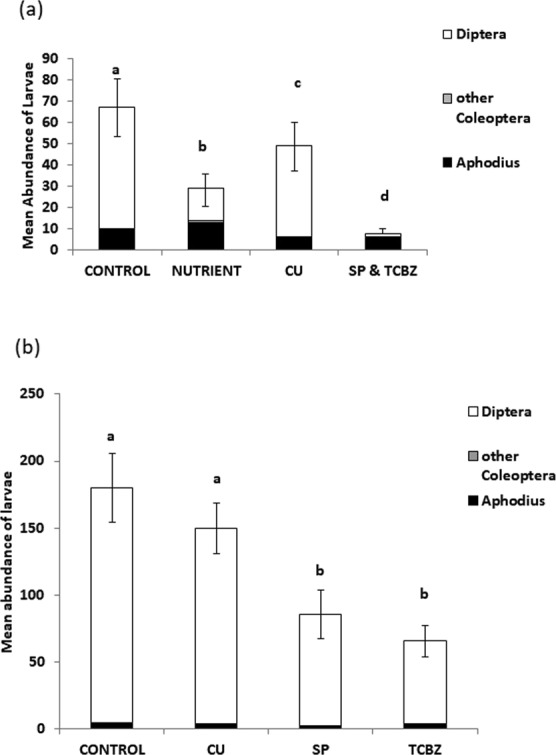
Mean abundance (and standard error on total mean abundance rather than individual species groups) of invertebrate larvae from experimental faecal pat cores and proportions of each of three types: *Diptera*, *Aphodius* and other *Coleoptera* larvae, in (**a**) 2014 and (**b**) 2015 for each treatment type at each time of sampling. Significant differences (from post hoc LSM tests in GLMMX analyses) between treatments in the mean abundance of larvae are represented by letters, where bars that do not share letters differ significantly from each other. The overall GLMMX significance of treatment effect is given in Table 1.

**Figure 3:**
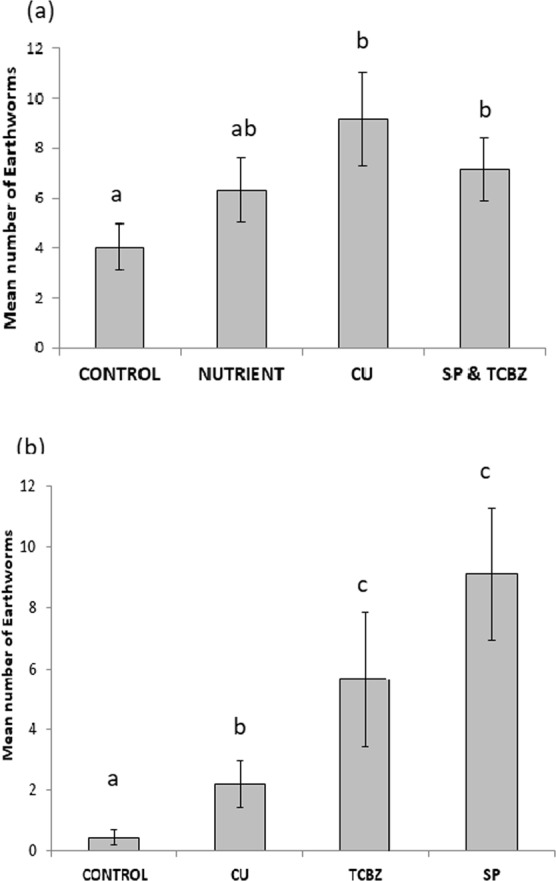
The mean (and standard error) abundance of earthworms in whole experimental faecal pats at week 4, in (**a**) 2014 and (**b**) 2015. The mean number of earthworms is compared between treatment types. Significant differences (from post hoc LSM tests in GLMMX analyses) between treatments are represented by letters, where, bars that do not share letters are significantly different. In each year, there were 27 experimental faecal pats of each treatment type, see the methods for the random block design used.

In 2014, 309 double core samples were taken across weeks 1, 2 and 4 from 103 experimental faecal pats, and in 2015, 324 double core samples were taken across weeks 1, 2 and 4 from 108 experimental faecal pats. A total of 1412 and 5251 larvae were counted in all of the cores, and 3981 and 12994 larvae were counted in all of the whole experimental faecal pats in 2014 and 2015 respectively. Within whole experimental faecal pats sampled in week four there were significantly fewer larvae in the TCBZ and SP dung (Table 1); and this effect was stronger and more consistent on *Diptera* larvae (Fig. 2 shows results from 2014 and Fig. 3 shows 2015 data). In 2014 there were significantly fewer *Diptera* larvae (*t* = 23.08, *P* < 0.001, *DF* = 50) and there was a trend to fewer *Aphodius* larvae (*t* = 1.98, *P* = 0.054, *DF* = 65.01) in the TCBZ and SP dung, compared to the CONTROL dung; with the largest reduction being in the number of *Diptera* (86% fewer on average). There was also a significant negative effect of COPPER on *Aphodius* larvae in 2014 (*t* = 2.25, *P* = 0.028, *DF* = 67.3), but the COPPER treatment did not significantly reduce the number of *Diptera* larvae in 2014 (*t* = 0.72, *P* = 0.092, *DF* = 49); and in 2015 there was no significant reduction in *Diptera* (*t* = 0.18, *P* = 0.86, *DF* = 87.3) or *Aphodius* larvae (*t* = 0.81, *P* = 0.42, *DF* = 78.6) with COPPER treatment. In 2015, there was a significant reduction in the number of *Diptera* larvae (64% fewer on average) in dung from cattle treated with SP (*t* = 4.55, *P* < 0.0001, *DF* = 87.4), and TCBZ (*t* = 5.02, *P* < 0.0001, *DF* = 84.4), (Fig. 3), but no significant overall treatment reduction in *Aphodius* larvae (*F* = 1.2, *P* = 0.33, *DF* = 3, 85.4). In the core samples, there was a significant effect of treatment on larval abundance in both years (Table 1). There was a significant effect of week (2014: *F* = 241.2, *DF*_*level*_ = 2,1, *P* = 0.045; 2015: *F* = 104, *DF*_*level*_ = 2,1, *P* < 0.001*)*, with no interaction between week and treatment in 2014 (*F* = 4.81, *DF*_*level*_ = 6,1, *P* = 0.336), but a significant interaction in 2015 (*F* = 22.3, *DF*_*level*_ = 6,1, *P* = 0.0161). The effect of treatment was not apparent in core samples from week 1, only in weeks 2 and 4, when there were fewer larvae in cores sampled from the dung collected from animals treated with SP and TCBZ, both when these treatments were combined in 2014, and when they were given separately in 2015. Independent of treatment effect, the number of larvae increased after week-1 in all cases, (see online Supplementary Figs S1a,b and S2a,b, for arthropod abundance by treatment and sampling week).

### Arthropod adults

We counted 747 and 141 adult arthropods across all weeks in all cores in 2014 and 2015 respectively; 48 and 46 adults were recorded in week 4 in all whole experimental faecal pats in 2014 and 2015. There were so few adult arthropods in the experimental dung that GLMMX analyses of treatment effects were only performed on the relatively larger numbers recorded within core samples in 2014, in which there was no overall treatment effect (Table 1). The mean abundance of adults in the experimental faecal pats, both in cores and in the whole experimental faecal pats in 2014 and 2015 are shown in Supplementary Figs S2a,b and S3a,b.

Post hoc weather data^32^ for Islay for the 4-week sampling period in both years was: mean temperature °C, 2014 (13.7, 4–19), 2015 (12.2, 4–18); relative (%) humidity, 2014 (86, 55 minimum), 2015 (86, 56 minimum); mean wind speed m/s, 2014 (4.5), 2015 (5.9); rainfall days, 2014 (6.1) 2015 (7.4).

### Oligochaeta (earthworms) and natural faecal pats

There was a significant positive effect of all livestock health treatments on earthworm abundance relative to controls in both years (Table 1). There were more earthworms in dung of animals treated with COPPER (2014: t = 3.76, DF = 104, P = 0.0004; 2015: t = 3.68, DF = 104, P = 0.0004), TCBZ (2015: t = 5.43, DF = 104, P < 0.0001),SP (2015: t = 7.22, DF = 104, P < 0.0001) and combined TCBZ&SP (2014: t = 2.91, DF = 104, P = 0.0054), than in CONTROL dung, in the whole experimental faecal pats at week 4 (Figs 3a,b).

### Comparison of natural and experimental faecal pats

On average, there were more larval and adult arthropods in the control experimental faecal pats than there were in the natural faecal pats produced by cattle in the fields surrounding the experimental grids (Table 1).

## Discussion

This is the first reported study to show that the commonly used animal health treatments for cattle, triclabendazole and deltamethrin, when used in the field as directed by the manufacturers, have the potential to significantly reduce arthropod larvae in faeces. The study was intended to provide a controlled and replicated evaluation in the natural environment of the effects of commercially available TCBZ and SP treatments on invertebrate density, as they are routinely applied to cattle at pasture on Islay. It provides evidence of an adverse effect of both these products on the density of arthropod larvae in faeces. The study also suggests that Cu-containing boluses have the potential to influence arthropod larval density in faeces. Whereas the density of arthropod larvae was reduced by treatment of animals with TCBZ and SP, there was a significant increase in the density of earthworms. All of these results are consistent with previous literature^11,20,26,28^, although this is the first report in which the effects of both SP and TCBZ have been documented in the field, rather than in controlled, laboratory settings.

To achieve a replicated, controlled study it was necessary to pool and freeze faecal samples from several treated animals, to construct artificial, experimental faecal pats, and to arrange these pats in a grid pattern with protection from livestock and birds. We found more arthropod larvae and adults in the same volume of faeces taken from control experimental faecal pats than we did from natural faeces of a similar age in the same or adjacent fields. This showed that the collection, pooling, freezing and presentation of faeces did not adversely affect faecal utilisation by invertebrates. We did not measure the concentration of the active compounds or their metabolites, nor have knowledge of their exact date of deposition, nor complete information on the treatments used on all of the livestock that produced the natural faecal pats, so we cannot say what caused the relative paucity of arthropods.

There was no initial difference in the numbers of dipteran adults that were observed to land on experimental faecal pats from differently treated cattle; however, it was dipteran larvae that were most significantly reduced in faeces from treated cattle. Whether the reduced number of invertebrates in faeces in animals treated with SP and TCBZ was a consequence of avoidance by adults of the faeces from treated cattle for oviposition, or a consequence of egg or larval mortality cannot be stated with certainty. The method we used for observing and counting adult invertebrates attracted to faecal pats was quite limited. However, there was no significant difference among treatments in the apparent attractiveness of the dung and in previous oviposition experiments, female sepsid flies were shown not to discriminate against dung that contained toxic ivermectin residues^35^. We therefore infer that the differences in density of larval arthropods in faeces from animals exposed to the treatments in our study were most likely a consequence of impaired development and survival in faeces from treated animals rather than reduced attractiveness to ovipositioning adults of faeces from cattle exposed to the treatments.

The timing of treatments in relation to the time of collection of faecal samples is important in the interpretation of and extrapolation from the observed effects. TCBZ is rapidly metabolised to the active molecule TCBZ-SO and the inactive TCBZ-SO_2_, and studies in ruminants suggest that the concentrations of TCBZ-SO and TCBZ-SO_2_ peak in plasma at about 40 h post-administration, with TCBZ-SO declining to pre-treatment concentrations at about 100 h and TCBZ-SO_2_ remaining high for over 140 h in cattle and buffalo^25,36^. Although we were unable to find published reports of the disposition of TCBZ in ruminant faeces, the official UK summary of product characteristics (SPC) for Fasinex states that “Triclabendazole sulfoxide reaches peak concentrations approximately 1 day after administration of FASINEX and the sulfone reaches peak concentrations approximately 3 days after administration. Both metabolites bind strongly to plasma protein, particularly albumin. Metabolites are excreted via the bile, primarily as conjugates. More than 90% of the total dose of FASINEX is excreted in the faeces, about 5% in the urine and 1% in milk. Elimination is virtually complete by 10 days after administration.”^37^. The interval of 4–5 days after administration of TCBZ that was selected for the collection of faeces is justified by this information. Similar information regarding the disposition of deltamethrin in faeces is not provided for veterinary products in the UK; however, there are several relevant peer-reviewed journal reports. Faeces appears to be the main route of excretion of deltamethrin in cattle^38^, and most of the deltamethrin recovered from faeces of cattle after oral administration was un-metabolized deltamethrin^38,39^, with peak values occurring two to four days post administration^38^. After the application of pour-on deltamethrin to dairy cattle, deltamethrin was detected in blood and in faeces within 9 hours^27^. Faecal concentrations increased up until day-4 and remained high until the conclusion of the study at day-8^27^. On this basis, our chosen sampling time in relation to treatment with deltamethrin is justified.

Copper is well known to have insecticidal capability^40^ and copper-containing boluses administered to sheep and cattle lodge in the reticulum and gradually release copper over several months. It might therefore be expected that faeces from cattle with copper-containing boluses would harbour fewer arthropod larvae than from untreated cattle. However, previous work in the UK did not show consistent negative effects of treating cattle with copper-containing boluses^23^. Similarly, we did not find consistent negative effects of copper-containing boluses although there was some evidence of a reduction in abundance of specific taxa on some occasions.

We saw a large difference in the abundance of flies between years, consistent with previous reports^12,41,42^, mainly related to variation in weather conditions. For example, the severity of the winter affects survival of adult flies^41^ and of larvae and pupae^43^. Despite the difference in general abundance of flies between years, we saw a strong effect of treatment. We cannot explain the low number of adult arthropods that were found in dung during the study. There was no suggestion from our own, nor officially recorded weather data for the setting out and sampling periods, that the weather specifically during these times was unusual or differed between these years.

The significant increase in earthworms found in some treated faecal samples might be caused by either unbalanced competition, particularly from reduced numbers of dung beetles^44,45^, or by changes in the microbial communities in the faeces of animals treated with SP products, as reviewed elsewhere^24^. The increased densities of faecal *Salmonella* spp., coliforms, heterotrophic and proteolytic bacteria that have been reported in slurry from SP-treated faeces might be to the advantage of earthworms.

Our primary reason for studying arthropod density in livestock faeces was their importance as a source of food for the rare and declining chough. At the north-western edge of the species’ European breeding range on Islay, Oronsay and Colonsay, population change is driven primarily by variation in pre-breeding survival (especially first-year) during which younger birds gather in socially structured flocks^46,47^. Here, first-year survival has been unprecedentedly low since 2007, causing population decline and threatening population viability^2^, but late-summer food supplementation has been shown to improve survival rates^3^. The definitive cause of the sudden and severe drop in first-year survival rates remains undiagnosed, but the response to food supplementation suggests that food shortage may be important. McCracken *et al*. highlighted the importance to chough of seasonally available prey^48^. In late summer, dung invertebrates, including beetle and fly larvae, are an important food source^33,48–50^, perhaps even more so to young chough^51^.

Studies of the impact on faecal arthropods of parasiticides applied to livestock are inherently controversial and likely subject to criticism by pharmaceutical companies on the one hand and advocates for environmental preservation on the other. The need for a methodical approach to the determination of environmental risks of parasiticide application has been concisely stated by Forbes in 1996^52^. In the tiered testing system he discussed, the first tier comprises laboratory-based dose-response bioassays; the second comprises “pilot field studies using methods more closely related to likely practical usage”; the third comprises “large scale trials under commercial use conditions carried out over extended […] time periods.” According to this hierarchy, our study fits into the second tier. Because of financial limitations we were unable to measure the concentrations of active ingredients or copper within the experimental or natural faecal pats, and therefore we cannot definitively conclude that the effects of treatments applied to the animals in the study were direct effects of the active ingredients that were applied. It is also recognised that the applications of copper had the potential to confound the applications of TCBZ and SP, both parasiticides being administered during the period in which the copper bolus would be actively leaching into the gut. It is precisely these potentially interacting trial-specific and location-specific factors that make the third tier, large scale studies essential.

The timing of treatments with animal health products is important when considering their potential cumulative impact on faecal arthropod densities^53^. If livestock are housed immediately after a treatment, then their faeces is stored in slurry pits or in manure piles for extended periods before being spread on pasture. This would be expected to reduce any impact of TCBZ administered during housing on arthropod communities. However, removing cattle from the fields completely eliminates the possibility of deposition of any new faecal pats on the pasture, presenting a greater problem with respect to faecal arthropod availability for birds. In contrast to treatments with TCBZ, SP treatments for ticks are almost exclusively applied when livestock are at pasture because this is the only time that they are at risk of infestation.

## Conclusion

This study demonstrated a consistent and significant reduction in the abundance of arthropod larvae in experimental faecal pats made from faeces of cattle that had been treated with triclabendazole and deltamethrin, alone and in combination, when used as directed by the manufacturers and under field conditions. The abundance of earthworms in experimental faecal pats from treated cattle at 4 weeks after exposure to the environment was significantly higher than in experimental pats from untreated cattle. The study did not detect any consistent effect of the administration of copper-containing boluses on arthropod larvae. The arthropods that were reduced by the triclabendazole and deltamethrin treatments are known to be a feed source for the locally endangered red-billed chough.

## Supplementary information

Supplementary Figures

## Data Availability

The datasets analysed during the current study are available from the corresponding author on reasonable request.

## References

[1] Bignal EM, McCracken DI (1996). Low-intensity farming systems in the conservation of the countryside. J. Appl. Ecol..

[2] Reid JM (2011). Diagnosing the timing of demographic bottlenecks: sub adult survival of red-billed choughs. J. Appl. Ecol..

[3] Bignal E, Bignal C (2011). Supplementary feeding of subadult Choughs. Brit. Wildl..

[4] Wall R, Strong L (1987). Environmental consequences of treating cattle with the antiparasitic drug ivermectin. Nature.

[5] Suarez VH, Lilschitcz ALL, Sallovitz JM, Lanusse CE (2003). Effects of ivermectin and doramectin faecal residues on the invertebrate colonization of cattle dung. J. Appl. Entomol..

[6] Adler N (2016). Effects of ivermectin application on the diversity and function of dung and soil fauna: Regulatory and scientific background information. Environ. Toxicol. Chem..

[7] Bai SH, Ogbourne S (2016). Eco-toxicological effects of the avermectin family with a focus on abamectin and ivermectin. Chemosphere..

[8] Floate KD, Spooner RW, Colwell DD (2001). Larvicidal activity of endectocides against pest flies in the dung of treated cattle. Med. Vet. Entomol..

[9] McCracken DI (1992). The potential for avermectins to affect wildlife. Vet. Parasitol..

[10] McCracken DI, Foster GN (1993). The effect of ivermectin on the invertebrate fauna associated with cow dung. Environ. Toxicol. Chem..

[11] Beynon SA, Wainwright WA, Christie M (2015). The application of an ecosystem services framework to estimate the economic value of dung beetles to the UK cattle industry. Ecol. Entomol..

[12] Webb L, Beaumont DJ, Nager RG, McCracken DI (2007). Effects of avermectin residues in cattle dung on yellow dung fly *Scathophaga stercoraria* (Diptera: *Scathophagidae*) populations in grazed pastures. Bull. Entomol. Res..

[13] Webb L, Beaumont DJ, Nager RG, McCracken DI (2010). Field-scale dispersal of *Aphodius* dung beetles (*Coleoptera: Scarabaeidae*) in response to avermectin treatments on pastured cattle. Bull. Entomol. Res..

[14] European Commission *Rural Development Programme – Scotland. 2014–2020*. V 2.2European Commission. 824pp. http://www.gov.scot/Resource/0050/00501661.pdf (accessed 05.06.17) (2016).

[15] Perry, B. & Sones, K. Global livestock disease dynamics over the last quarter century: drivers, impacts and implications. Rome, Italy: FAO; (Background paper for the SOFA 2009) (2009).

[16] Mas-Coma S, Valero MD, Bargues MD (2009). Climate change effects on trematodiases, with emphasis on zoonotic fascioliasis and schistosomiasis. Vet. Parasitol..

[17] Van Dijk J, Sargison ND, Kenyon F, Skuce PJ (2010). Climate change and infectious disease: helminthological challenges to farmed ruminants in temperate regions. Animal..

[18] Gordon D, Zadoks R, Skuce P, Sargeson N (2012). Confirmation of triclabendazole resistence in liver fluke in the UK. Vet. Rec..

[19] Foil LD (2004). Factors that influence the prevalence of acaricide resistance and tick-borne diseases. Vet. Parasitol..

[20] Mann CM, Barnes S, Offer B, Wall R (2015). Lethal and sub-lethal effects of faecal deltamethrin residues on dung-feeding insects. Med. Vet. Entomol..

[21] Vale GA, Hargrove JW, Chamisa A, Grant IF, Torr SJ (2015). Pyrethroid Treatment of Cattle for Tsetse Control: Reducing Its Impact on Dung Fauna. Negl. Trop. Dis..

[22] Beynon SA, Mann DJ, Slade EM, Lewis OT (2012). Species-rich dung beetle communities buffer ecosystem services in perturbed agro-ecosystems. J. Appl. Ecol..

[23] Beynon SA, Peck M, Mann DJ, Lewis OT (2012). Consequences of alternative and conventional endoparasite control in cattle for dung-associated invertebrates and ecosystem functioning. Agric. Ecosys. Environ..

[24] Beynon SA (2012). Potential environmental consequences of administration of ectoparasiticides to sheep. Vet. Parasitol..

[25] Mestorino N (2008). Pharmacokinetic disposition of triclabendazole in cattle and sheep; discrimination of the order and the rate of the absorption process of its active metabolite triclabendazole sulfoxide. Vet. Res. Comm..

[26] Kılıç A, Büyükgüzel K, Büyükgüzel E (2015). The effect of anthelmintic triclabendazole on survival and development of *Galleria mellonella (Lepidoptera: Pyralidae*) L. rearing on artificial diet. Kafkas Üniversitesi Veteriner Fakültesi Dergisi.

[27] Venant A, Belli P, Borrel S, Mallet J (1990). Excretion of deltamethrin in lactating cattle. Food Addit. Contam..

[28] Wardhaugh KG, Longstaff BC, Lacey MJ (1998). Effects of residues of deltamethrin in cattle faeces on the development and survival of three species of dung-breeding insect. Aust. Vet. J..

[29] Sommer C, Jensen KMV, Jespersen JB (2001). Topical treatment of calves with synthetic pyrethroids: effects on the non-target dung fly *Neomyia cornicina* (Diptera: Muscidae). Bull. Entom. Res..

[30] Sands, B., Mgidiswa, N., Nyamukondiwa, C. & Wall, R. Environmental consequences of deltamethrin residues in cattle faeces in an African agricultural landscape.*Ecol. and Evol*. **8**, 2938–2946 (2018). 10.1002/ece3.3896 PMC5838066 29531707

[31] Yamashita N, Hayakawa H (1991). Reproduction of a dung beetle, *Onthophagus gazella*, fed with frozen dung of pastured cattle. Japan Soc. Med. Entom. Zool..

[32] Met Office. Met Office Integrated Data Archive System (MIDAS) Land and Marine Surface Stations Data (1853-current). NCAS British Atmospheric Data Centre, 5th Feb 2019. http://catalogue.ceda.ac.uk/uuid/220a65615218d5c9cc9e4785a3234bd0 (2012).

[33] McCracken DI, Foster GN (1994). Invertebrates, cow-dung, and the availability of potential food for the chough (*Pyrrhocorax pyrrhocorax* L.) on pastures in north-west Islay. Environ. Conserv..

[34] SAS Institute Inc., 2015. *SAS® 9.4 Statements: Reference*, Fourth Edition. Cary, NC: SAS Institute Inc.

[35] Blanckenhorn WU, Puniamoorthy N, Schäfer MA, Scheffczyk A, Römbke J (2013). Standardized laboratory tests with 21 species of temperate and tropical sepsid flies confirm their suitability as bioassays of pharmaceutical residues (ivermectin) in cattle dung. Ecotoxicol. Environ. Saf..

[36] Sanyal PK, Gupta SC (1996). Efficacy and pharmacokinetics of triclabendazole in buffalo with induced fasciolosis. Vet. Parasit..

[37] VMD (Veterinary Medicines Directorate. Product Information Database http://www.vmd.defra.gov.uk/ProductInformationDatabase/Default.aspx - accessed on 07/03/2018 (2018).

[38] Akhtar MH, Hartin KE, Trenholm HL (1986). Fate of [C14] deltamethrin in lactating dairy cows. J. Agric. Food Chem..

[39] Croucher A, Hutson DH, Stoydin G (1985). Excretion and residues of the pyrethroid insecticide cypermethrin in lactating cows. *Pest Man*. Science.

[40] Dow JAT (2017). The essential roles of metal ions in insect homeostasis and physiology. Current Opinion in Insect Science.

[41] Gibbons DS (1987). The causes of seasonal changes in numbers of the yellow dung fly, *Scathophaga stercoraria* (Diptera: *Scathophagidae*). Ecol. Entom..

[42] Ward PI, Simmons LW (1990). Short‐term changes in numbers of the yellow dung fly *Scathophaga stercoraria* (Diptera: *Scathophagidae*). Ecol. Entom..

[43] Pitts KM, Wall R (2006). Cold shock and cold tolerance in larvae and pupae of the blow fly, *Lucilia sericata*. Physiol. Entomol..

[44] Hirschberger P, Degro HN (1996). Oviposition of the dung beetle *Aphodius ater* in relation to the abundance of the yellow dungfly *Scatophaga stercoraria*. Ecol. Entomol..

[45] O’Hea NM, Kirwan L, Finn JA (2010). Experimental mixtures of dung fauna affect dung decomposition through complex effects of species interactions. Oikos.

[46] Still E, Monaghan P, Bignal EM (1987). Social structuring at a communal roost of Choughs *Pyrrhocorax pyrrhocorax*. Ibis.

[47] Reid JM, Bignal EM, Bignal S, McCracken DI, Monaghan P (2004). Identifying the demographic determinants of population growth rate: a case study of red-billed choughs *Pyrrhocorax pyrrhocorax*. J. Anim. Ecol..

[48] McCracken DI, Foster GM, Bignal EM, Bignal S (1992). An assessment of chough *Pyrrhocorax pyrrhocorax* diet using multivariate analysis technique. Avocetta..

[49] Warnes, J. & Stroud, D. A. Habitat use and the diet of Chough on the island of Islay Scotland. In: Choughs and land-use in Europe, Proceedings of an international workshop on the conservation of the chough, *Pyrrhocorax pyrrhocorax*, in the EC. 11–14 November 1988, 112 pp, ISBN 0 9515038 0 4 pp 46–51 (1989).

[50] McKay, C. R. Conservation and ecology of the red-billed chough Pyrrhocorax pyrrhocorax. *Doctoral dissertation, University of Glasgow* (1996).

[51] McCracken DI, Bignal EM (1998). Applying the results of ecological studies to land-use policies and practices. J. Appl. Ecol..

[52] Forbes AB (1996). Environmental assessments in veterinary parasitology: A balanced perspective. Int. J. for Parasit..

[53] Wratten SD, Forbes AB (1996). Environmental assessment of veterinary avermectins in temperate pastoral ecosystems. Ann. of Appl. Biol..

