# Supplementary material for: Adverse effects of routine bovine health treatments containing triclabendazole and synthetic pyrethroids on the abundance of dipteran larvae in bovine faeces
Source: Sci Rep. 2019 Mar 13;9:4315. doi: 10.1038/s41598-019-40800-6 (PMC6416274; doi:10.1038/s41598-019-40800-6)

Adverse effects of routine bovine health treatments containing triclabendazole and synthetic pyrethroids on the abundance of dipteran larvae in bovine faeces

Gillian Gilbert[a] *, Fiona S MacGillivray[b] Helen L Robertson[c] and Nicholas N Jonsson[d]

[a, c] Royal Society for the Protection of Birds, Centre for Conservation Science, 10 Park Quadrant, Glasgow, G36BS, Scotland, UK.

[b] Royal Society for the Protection of Birds, Bushmill Cottage, Gruinart, Bridgend Isle of Islay, Argyll, PA44 7PR, Scotland, UK.

[d] University of Glasgow College of Medical, Veterinary and Life Sciences, 464 Bearsden Rd Glasgow G61 1QH, Scotland UK.

***Corresponding author, email address:** gillian.gilbert@rspb.org.uk.

Supplementary Figures 1a, 1b, 2a, 2b, 3a & 3b.

Supplementary Figure Descriptions

Supplementary Figure 1 a & b The effect of four cattle treatments including a control, on the mean abundance of larval invertebrates in standard cores sampled from experimental cow pats, in weeks 1, 2 and 4 of being laid out in a random block design. Mean (and standard error on total mean abundance rather than individual species groups) abundance of larval invertebrates per standard core sample and proportions of each of three groups:

Diptera, *Aphodius* and other Coleopteran larvae, for a) 2014 and b) 2015. Significant differences (from post hoc LSM tests in GLMMX analyses) between treatments in the total abundance of larvae within each week are represented by letters, where, within each week, bars that do not share letters have significantly different means. The overall GLMMX significance of treatment effect within weeks is given in Table 2. There were 27 experimental dung pats of each treatment type, see the methods for the random block design used.

Supplementary Figure 2a & b The effect of four cattle treatments including a control, on the mean abundance of adult invertebrates in standard cores sampled from experimental cow pats, in weeks 1, 2 and 4 of being laid out in a random block design. Mean (and standard error on total mean abundance rather than individual species groups) abundance of adult invertebrates per standard core sample and proportions of each groups, for a) 2014 and b) 2015. For a) 2014, the overall GLMMX significance of treatment effect within weeks is given in Table 2, and significant differences (from post hoc LSM tests in GLMMX analyses) between treatments in the total abundance of adults within each week are represented by letters, where, within each week, bars that do not share letters have significantly different means. There were not enough adult invertebrates recorded in 2015 to perform GLMMX analyses. There were 27 experimental dung pats of each treatment type, see the methods for the random block design used.

Supplementary Figure 3 a and b Mean (and standard error on total mean abundance rather than individual species groups) abundance of adult invertebrates from whole experimental dung pats and proportions of each type present, for a) 2014 and b) 2015. The mean number of adults per experimental dung pat of each treatment type, after week 4. There were not enough adult invertebrates recorded in whole experimental dung pats to perform GLMMX analyses. There were 27 experimental dung pats of each treatment type, see the methods for the random block design used.

Supplementary Figure 1 a and b

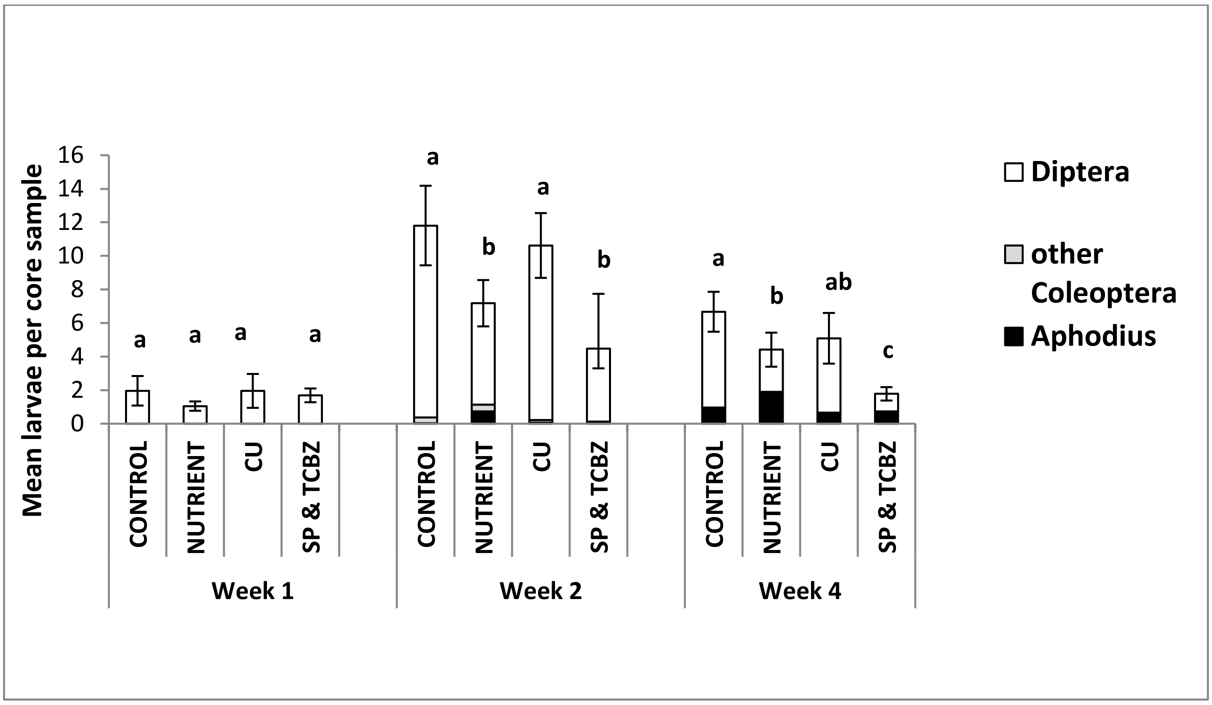

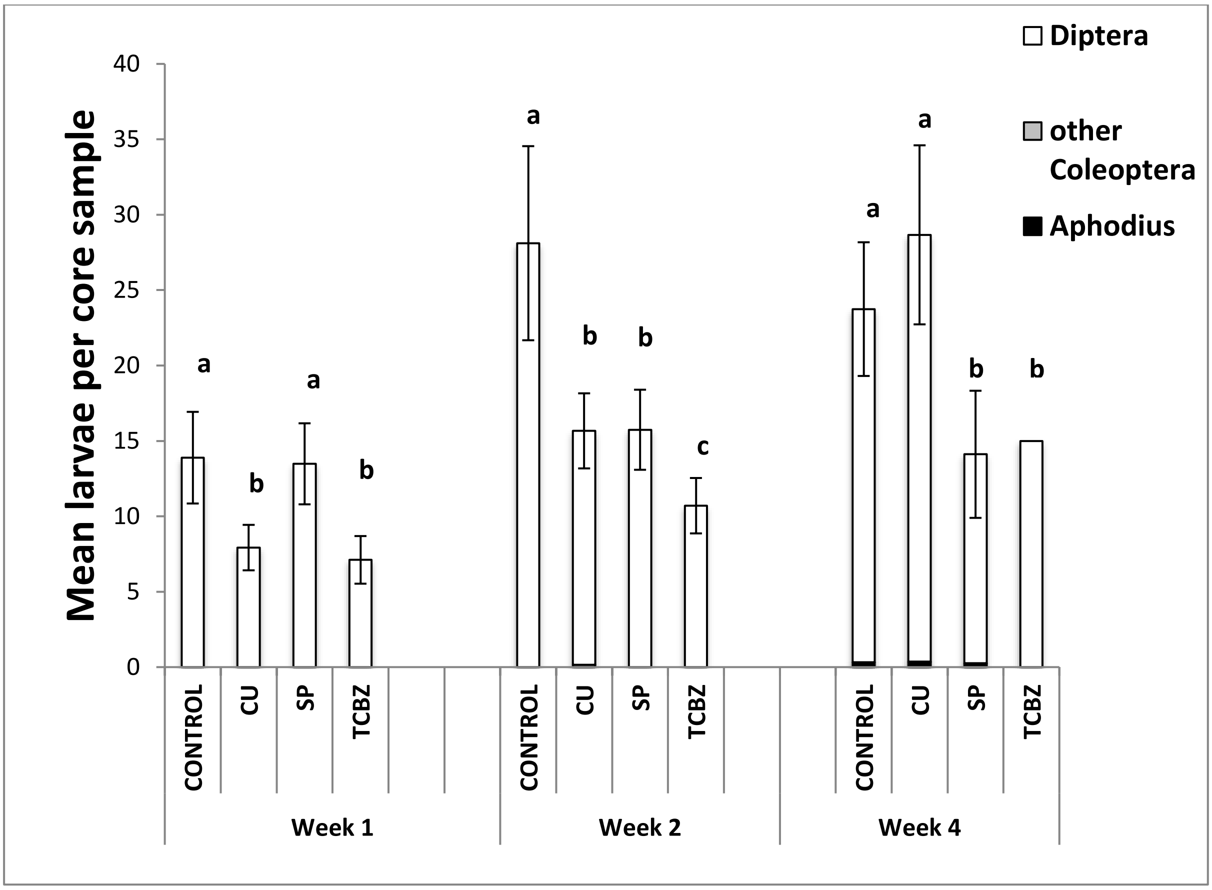

Supplementary Figure 2 a & b

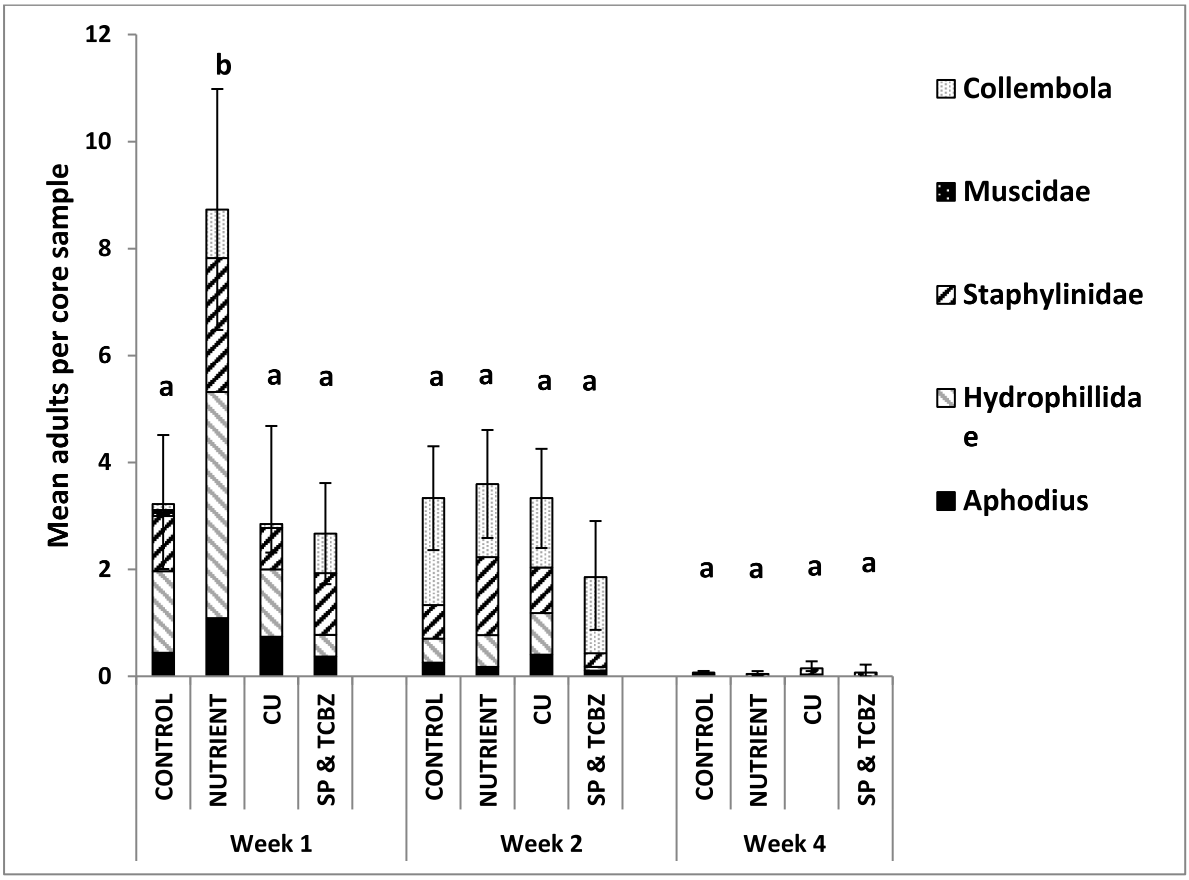

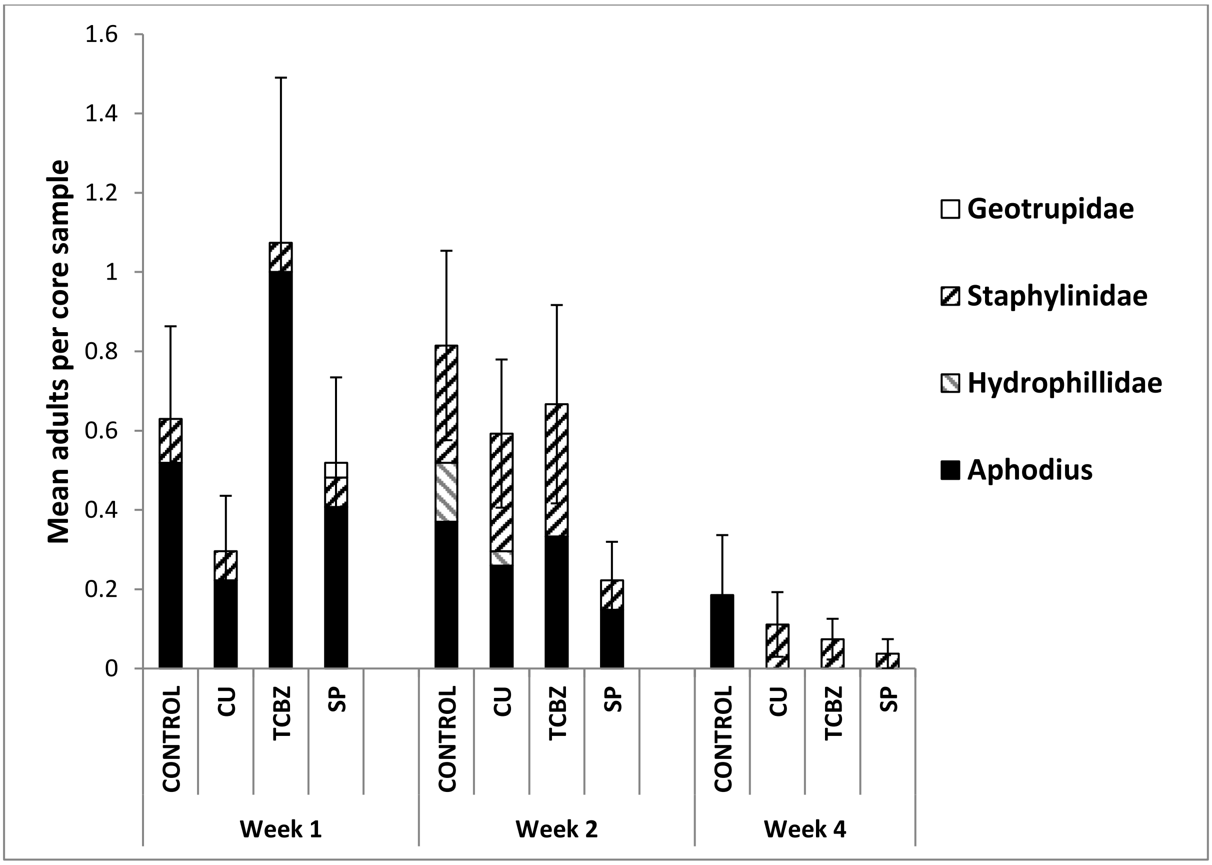

Supplementary Figure 3 a & b

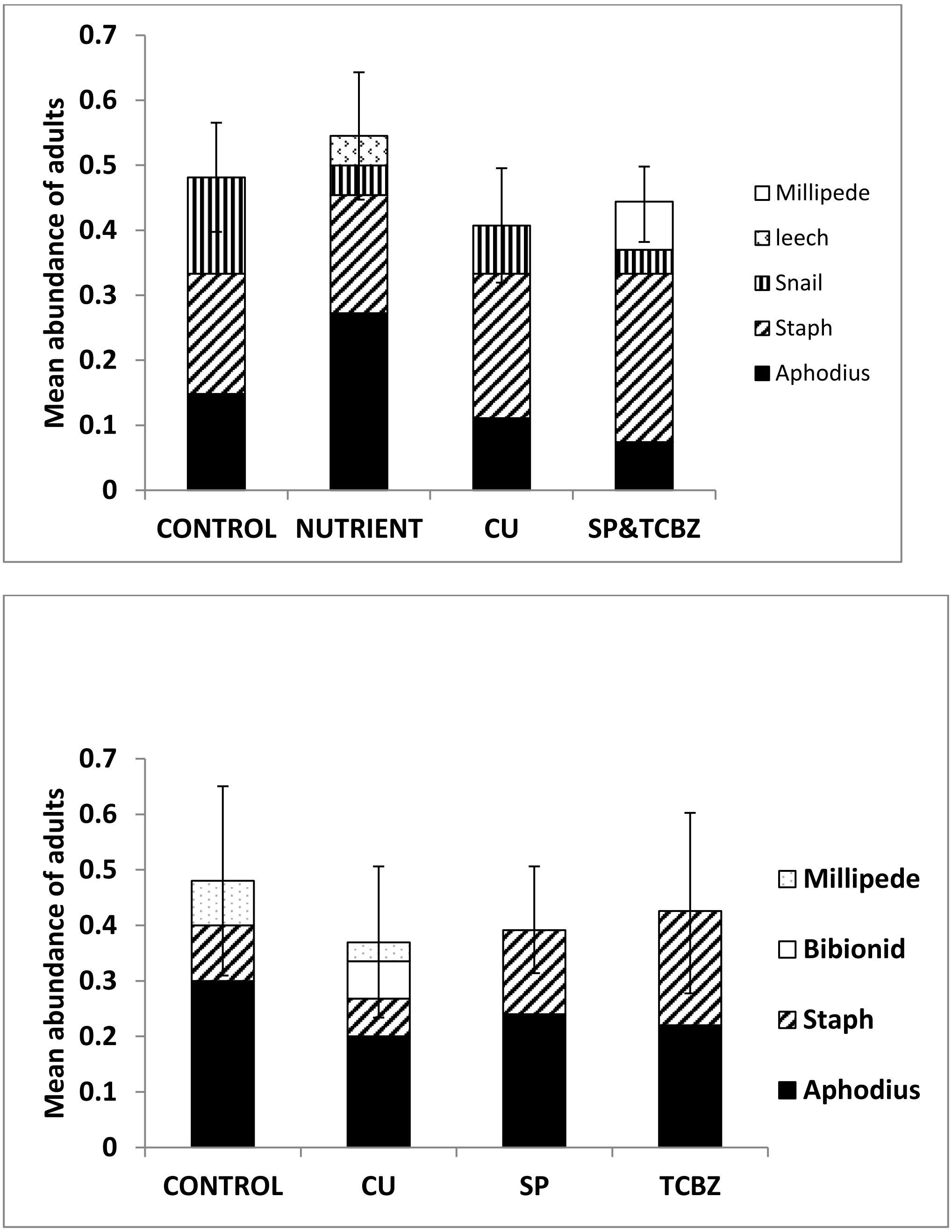

Supplement: Supplementary file 1 — Supplementary Figures [file 41598_2019_40800_MOESM1_ESM.pdf]
